# Influence of birth rates and transmission rates on the global seasonality of rotavirus incidence

**DOI:** 10.1098/rsif.2011.0062

**Published:** 2011-04-20

**Authors:** Virginia E. Pitzer, Cécile Viboud, Ben A. Lopman, Manish M. Patel, Umesh D. Parashar, Bryan T. Grenfell

**Affiliations:** 1 Department of Ecology and Evolutionary Biology, Princeton University, Princeton, NJ 08544, USA; 2 Woodrow Wilson School of Public and International Affairs, Princeton University, Princeton, NJ 08544, USA; 3 Fogarty International Center, National Institutes of Health, Bethesda, MD 20892, USA; 4 Epidemiology Branch, Division of Viral Diseases, National Center for Immunization and Respiratory Diseases, Centers for Disease Control and Prevention, Atlanta, GA 30033, USA

**Keywords:** rotavirus, seasonality, vaccination, infectious disease modelling

## Abstract

Rotavirus is a major cause of mortality in developing countries, and yet the dynamics of rotavirus in such settings are poorly understood. Rotavirus is typically less seasonal in the tropics, although recent observational studies have challenged the universality of this pattern. While numerous studies have examined the association between environmental factors and rotavirus incidence, here we explore the role of intrinsic factors. By fitting a mathematical model of rotavirus transmission dynamics to published age distributions of cases from 15 countries, we obtain estimates of local transmission rates. Model-predicted patterns of seasonal incidence based solely on differences in birth rates and transmission rates are significantly correlated with those observed (Spearman's *ρ* = 0.65, *p* < 0.05). We then examine seasonal patterns of rotavirus predicted across a range of different birth rates and transmission rates and explore how vaccination may impact these patterns. Our results suggest that the relative lack of rotavirus seasonality observed in many tropical countries may be due to the high birth rates and transmission rates typical of developing countries rather than being driven primarily by environmental conditions. While vaccination is expected to decrease the overall burden of disease, it may increase the degree of seasonal variation in the incidence of rotavirus in some settings.

## 1. Introduction

Rotavirus is one of the leading causes of severe diarrhoea in children in both developed and developing countries, and is estimated to cause over half a million deaths worldwide with much of this mortality burden concentrated in developing countries [1,2]. Two separate vaccines against rotavirus have been recently developed and licensed in many countries throughout the world. Early observations of vaccine impact in the USA, Australia and several countries in Latin America and Europe have highlighted the enormous promise such vaccines hold for preventing rotavirus-associated diarrhoea [3]. Clinical trials have estimated that vaccine recipients benefit from a 49–98% reduction in the risk of severe rotavirus diarrhoea depending on the setting, with lower efficacy being observed in low-income regions of Africa and Asia [4–10]. However, predicting the population-level impact of vaccination and the importance of herd immunity requires an understanding of the dynamics of infection. This can be achieved through mathematical modelling studies rooted in biological and epidemiological data [11–16].

The spatio-temporal patterning of disease can provide important insight into the underlying transmission dynamics of infections. Using a mathematical model for the transmission dynamics of rotavirus, we have previously shown that the apparent travelling wave of rotavirus infection in the USA from southwest to northeast may be due to underlying geographical variability in birth rates [13]. This pattern is no longer apparent in the post-vaccination era, suggesting that susceptible recruitment rather than environmental factors plays a key role in determining the timing of rotavirus activity in the US [17]. The model can also explain the much diminished and delayed peak in rotavirus activity observed during the 2007–2008 season following the licensing of the RotaTeq vaccine in the US in 2006, providing an important source of model validation [13,18,19]. Furthermore, the model allows quantification of the relative importance of direct and indirect (herd immunity) protection, which is essential in determining the long-term impact of vaccination [13]. However, these results are not directly applicable to the situation of developing countries. Routine immunization against rotavirus has only been introduced in developed countries, South Africa, and a handful of developing countries in Latin America thus far. While the majority of rotavirus deaths occur in the poorest nations, the impact of vaccination in such settings remains unclear.

There are several reasons why the dynamics of rotavirus infection may differ in developing countries compared with developed countries. Many developing countries are located in the tropics where traditionally rotavirus activity has been thought to lack seasonality, leading to high levels of year-round disease transmission [20]. However, recent country-level assessments of rotavirus epidemiology conducted in anticipation of rotavirus vaccination programmes suggest that this pattern may not be as universal as previously thought. Rotavirus tends to be more common in cooler, drier months in most settings, but seasonal peaks have been noted to occur year-round in different countries [21–23], and can vary over time in the same country [24]. Attempts to relate these patterns to climatic variables such as temperature, humidity and rainfall have led to conflicting results; it is possible the effect of certain climatic variables is context specific [25–28].

Birth rates have been shown to be an important driver of the dynamics of rotavirus in the US [13], and are likely influential in developing countries as well. Birth rates are often considerably higher in developing countries compared with developed countries, ranging as high as 45–50 live births per 1000 persons in parts of Asia and sub-Saharan Africa, compared with 8–20 per 1000 in developed countries [29]. Crowding and poor sanitation are also expected to increase rates of rotavirus transmission [30,31]. While temperature and humidity tend to exhibit less seasonal variation in developing countries in the tropics, other factors such as precipitation, population movements and birth rates may be more seasonally variable in such settings [32,33]. Our best-fit model to rotavirus hospitalization and laboratory data in the USA indicated that small seasonal changes in the transmission rate (approx. 5% seasonal amplitude) could explain the highly seasonal pattern of outbreaks [13]. Natural oscillations resulting from the replenishment of susceptible individuals through new births and/or waning of immunity—a phenomenon known as dynamical resonance—may be a key factor in explaining the seasonality of rotavirus, as in other diseases [34,35]. Overall, the environmental and demographic factors driving seasonality in rotavirus transmission have yet to be fully elucidated and quantified.

Here, we used a combination of mathematical models for rotavirus transmission and empirical data to examine how seasonal patterns of disease incidence vary with birth rates and mean transmission rates independent of variations in environmental factors. We tested the hypothesis that dynamic resonance can explain some or all of the observed geographical variation in rotavirus seasonality. By fitting our model of rotavirus transmission to the age distribution of rotavirus-associated diarrhoea cases from a variety of countries, we obtained estimates of mean transmission rates in these settings and validated predictions of rotavirus seasonality against observed data. We then explored how vaccination may impact seasonal patterns of rotavirus incidence if existing vaccines are introduced in more countries.

## 2. Methods

### 2.1. Epidemiological data

To obtain estimates of mean rotavirus transmission rates in different settings, we fit our model to published age distributions of rotavirus cases less than 5 years of age (an age range in which approx. 95% of reported cases occur) from 15 countries representing eight regions of the world [11,13,36–48]. To identify rotavirus surveillance studies which included a description of the seasonal pattern and age distribution of cases, we conducted a literature search using Web of Science. We limited our analysis to those studies that were published between 2005 and 2010 and reported the number of confirmed rotavirus diarrhoea cases by month (or number of tests and per cent of tests positive for rotavirus) over a period of at least 2 years, or for which such data were available from other sources [49–51]. We also required that the studies included data on either numbers or proportions of rotavirus-positive cases of acute gastroenteritis by age, with at least six month age resolution less than 1 year of age and 1 year age resolution up to 5 years of age. We used Digitizeit software (Bormann; www.digitizeit.de) to extract data from figures when the data were not available from tables. The quality and nature of rotavirus surveillance varies from country to country and across time; some studies tracked only hospitalized rotavirus cases, while others sampled both inpatient and outpatient populations. By examining the proportion of cases in each age class, we can control for differences in surveillance, provided biases in reporting are not related to the age of cases.

We obtained birth rate data from the World Population Prospects database [29] and assumed the birth rate in each country was constant over time and equal to the mean crude birth rate (CBR) for the 5 year periods prior to and including the study period. The background mortality rate was assumed to be equal to the birth rate and independent of age, so that the total population size remained constant. We ignored disease-induced mortality, which will not affect our estimates of the transmission rate provided mortality does not significantly shorten the infectious period.

### 2.2. Transmission model

Details of our age-structured model for the transmission dynamics of rotavirus are presented in the electronic supplementary material and have been described previously [13]. In short, we assume that susceptible individuals are infected, recover and are temporarily immune, then become susceptible again, with reduced susceptibility to infection and disease following one or more previous infections. We assume that only primary and secondary infections result in severe diarrhoea which is subsequently reported. After that, we assume individuals only experience asymptomatic (or mildly symptomatic) infections that are less infectious and not reported. After examining the age distributions of cases from different countries, we modified the model to more accurately capture the nature of maternal immunity (and/or limited exposure to rotavirus during the first 6 months of life due to social/behavioural factors). Maternal immunity appeared to be stronger than previously assumed, as indicated by a relative dearth of cases in the zero to five months age group, particularly in the lowest income countries. Thus, we split the maternal immunity compartment into six separate compartments and estimated a common duration of time spent in each compartment by fitting the model to the mean age distribution of cases from all countries combined (see the electronic supplementary material).

The rotavirus transmission rate was assumed to vary sinusiodally with a period of 1 year as follows: *β*(*t*) = *β*_0_(1 + *a* cos(2*π*(*t* − *φ*))), where, *β*_0_ is the baseline transmission rate, *a* is the amplitude of seasonality and *φ* is a seasonal offset parameter. Mixing was assumed to be homogeneous, such that *β*_0_ was the same for all age groups. We examined the sensitivity of our results to this assumption in the electronic supplementary material. The sinusoidal variation in the transmission rate is presumably related to environmental factors that could influence rotavirus transmission, such as the effect of temperature or humidity on virus survival or increased population crowding during cold or rainy months. However, the factors influencing this have yet to be fully elucidated. We varied the birth rate and baseline transmission rate while holding the amplitude of seasonal variation in the transmission rate, *a*, constant at 5 per cent, a level similar to that estimated for the USA. This allowed us to examine seasonality in the incidence of severe rotavirus diarrhoea predicted by the model *independent* of environmental factors. Other dynamic models for rotavirus have estimated the amplitude of seasonal variation in the transmission rate to be similar albeit slightly higher in England and Wales (*a* = 9.2%) [11] and Kyrgyzstan (*a* = 7.9%) [12]. If seasonal variation in the transmission rate is tied to factors such as temperature and humidity, however, it might be expected to exhibit lower amplitude in the tropics. We conducted sensitivity analyses with *a* = 2.5 and 10%, and examined the effect of modelling seasonal variation in the transmission rate using a step function (see the electronic supplementary material).

The degree of seasonal fluctuation in incidence (*s*) predicted by the model was measured as:
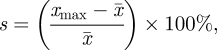
where *x*_max_ is the peak number and 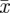 is the mean number of clinical rotavirus cases per week (in all age groups) over a 2 year period, to account for the possible occurrence of biennial epidemics. To quantify the observed seasonality, we first aggregated the data by month of the year then compared the mean rotavirus incidence in the three months surrounding the peak to the mean incidence for the entire 12 month period to limit the influence of stochastic variation in the number of rotavirus cases.

We calculated the basic reproductive number of primary infections, *R*_0_, and used it as a measure of transmissibility (see the electronic supplementary material). We first examined the dynamics of rotavirus epidemics predicted by the model at the observed birth rate and estimated *R*_0_ for each of the 15 countries, then across CBRs ranging from five to 50 live births per 1000 and *R*_0_ ranging from 20 to 100, consistent with most of the estimated global variation in these parameters (table 1).

**Table 1.** Summary of model parameters and seasonal patterns for 15 countries.

country (reference(s))	study years	CBR (live births per 1000)	estimated *R*_0_ (95% CI)	estimated seasonality, % (*rank*)	observed seasonality, % (*rank*)
*inpatient studies*					
Australia [43,50,51]	1997–2007	13.4	53.9 (52.2, 55.6)	54 (*10*)	104 (*10*)
Taiwan [48]	2005–2007	12.0	23.3 (20.4, 26.2)	140 (*14*)	102 (*9*)
USA [13]	1998–2004	14.8	45.3 (44.8, 45.8)	73 (*12*)	179 (*13*)
China [40]	2003–2007	13.7	88.1 (83.4, 93.4)	20 (*4*)	98 (*7*)
Nepal [46]	2005–2007	27.9	51.3 (43.2, 61.9)	25 (*5*)	100 (*8*)
Uzbekistan [41]	2005–2006	21.0	46.7 (42.4, 51.5)	40 (*8*)	63 (*5*)
Hong Kong SAR [44]	2001–2003	8.4	52.1 (48.7, 55.4)	145 (*15*)	132 (*11*)
Cambodia [45]	2005–2007	25.4	68.5 (62.0, 76.1)	16 (*3*)	46 (*4*)
Fiji [42]	2006–2007	22.1	46.2 (36.8, 58.6)	39 (*7*)	178 (*12*)
Lao PDR [36]	2005–2007	28.4	34.3 (30.4, 38.8)	49 (*9*)	241 (*15*)
*outpatient and inpatient studies*					
England and Wales [11,49]	1999–2009	12.0	54.4 (54.0, 54.8)	61 (*11*)	182 (*14*)
Bangladesh [47]	1993–2004	30.9	72.2 (68.4, 76.4)	11 (*2*)	42 (*2*)
Malawi [39]	1997–2007	45.1	191 (137, 313)	2 (*1*)	15 (*1*)
Nigeria [37]	2002–2004	42.7	37.0 (27.5, 51.9)	26 (*6*)	46 (*3*)
Philippines [38]	2005–2006	25.7	27.9 (24.4, 31.6)	110 (*13*)	94 (*6*)

### 2.3. Impact of vaccination

We then examined the impact of vaccination on patterns of rotavirus disease incidence predicted by the model. Vaccination was assumed to confer protection comparable to that of primary infection, as in our previous model [13], resulting in an 80 per cent reduction in the risk of severe diarrhoea in previously uninfected infants. Vaccine efficacy was not an input parameter, but this level of protection is similar to that estimated during Rotarix trials in Latin America [5]. Vaccine efficacy estimates in developing countries of Asia and Africa, however, have been lower [4,6,10]; this can be considered equivalent to a lower *effective* coverage level in our model. We examined the impact of vaccination at coverage levels of 50, 70 and 90 per cent on the mean and peak incidence of severe diarrhoea during a 2 year period beginning 5 years after the introduction of the vaccine across the full range of birth rates and transmission rates.

Finally, we examined model predictions regarding the proportion of infants infected prior to four months of age across the full range of birth rates and transmission rates and how this was impacted by vaccination. One possible explanation for the reduced efficacy of rotavirus vaccination in recent trials conducted in developing countries is the high rate of exposure to circulating virus in the placebo group prior to enrolment combined with limited immunogenicity of the vaccine in previously infected infants [6]. If vaccination mimics natural infection but does not provide any added benefit beyond the immunity generated by a *primary* rotavirus infection, or if recent infection interferes with vaccine-induced immunity, then it is possible that the observed vaccine effectiveness may be reduced in relation to the proportion of infants infected prior to the age of vaccine administration.

## 3. Results

**Figure 1.**
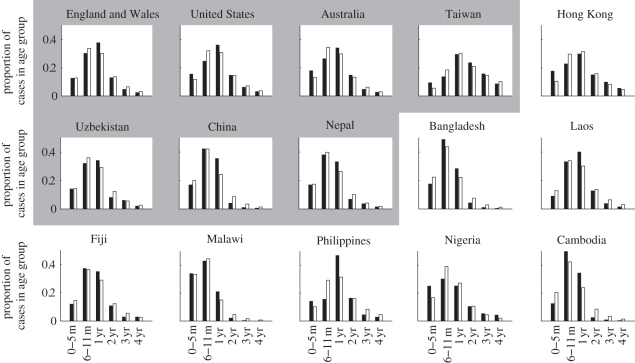
Fit of the model to age distributions of cases from 15 countries. The observed proportion of reported cases in the zero to five month, six to 11 month, 1, 2, 3 and 4 year old age groups are represented by the black bars, while the white bars are those predicted by the model. Developed countries are in the top line, while developing countries are in the bottom two lines. Within these groups, countries are ordered by distance from the equator (greatest to least); temperate countries are in the shaded region.

Fitting of the model to age distributions of cases from 15 countries yielded estimates of *R*_0_ ranging from 23.3 to 191 (table 1). Estimates of *R*_0_ were typically lower for more developed countries than they were for developing countries, where a high proportion of cases occurred in infants less than 1 year of age (figure 1). The degree of seasonal fluctuation in incidence observed was similar to that predicted by the model, although the magnitude of seasonal fluctuation was greater in the observed data than in the model predictions (table 1); this is not unexpected given that the stochasticity in the data will clearly affect this measure. If we increased the amplitude of seasonal forcing in the model to 10 per cent, we could better capture the observed magnitude of seasonal variation in most cases (see the electronic supplementary material, figure S3). Regardless of the amplitude of seasonal forcing, the observed and predicted seasonality ranks were significantly correlated (Spearman's correlation: *ρ* = 0.65, *p* < 0.05; table 1).

**Figure 2.**
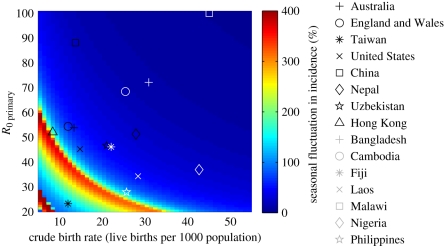
Predicted seasonal patterns of rotavirus incidence across a range of birth rates and transmission rates. The degree of seasonal fluctuation in incidence of rotavirus diarrhoea predicted by the model is indicated by the colour bar. The larger red–yellow region corresponds to strong annual epidemics, while the smaller red region in the lower left-hand corner corresponds to biennial epidemics. The estimated position for each of the 15 countries is plotted according to the symbols; note the estimated *R*_0_ for Malawi was greater than 100, but there was not much variation in the predicted seasonal pattern for this parameter region.

The degree of seasonal fluctuation in the incidence of severe rotavirus diarrhoea predicted by the model varied substantially depending on both the birth rate and transmission rate (figure 2 contour map). Seasonality, independent of the degree of seasonal forcing, was greatest for combinations of low-to-intermediate birth rates and low-to-intermediate transmission rates. A region of strong seasonality was also noted when both birth rates and transmission rates were very low (CBR < 10 live births per 1000 and *R*_0_ < 30); further exploration revealed that epidemics occurred biennially in this parameter region. When birth rates and transmission rates were high (e.g. CBR > 30 live births per 1000 and *R*_0_ > 50), the degree of seasonal fluctuation in incidence was predicted to be low (less than 25%). The parameter regions of strong seasonality varied only slightly with the strength of seasonal forcing (*a*) and were not sensitive to the functional form (sinusoidal or step), while the degree of seasonal fluctuation in incidence varied proportionally with *a*, as expected (see the electronic supplementary material).

**Figure 3.**
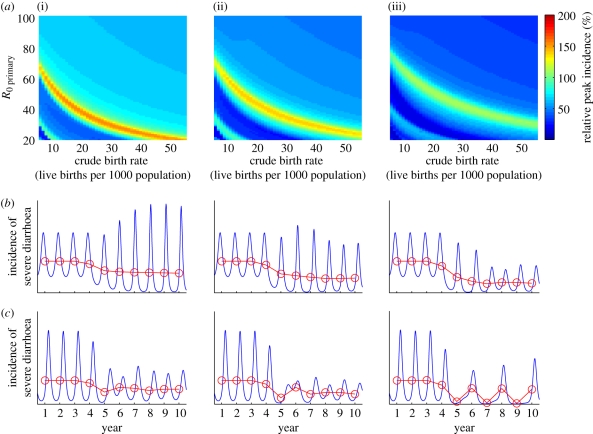
Predicted effect of vaccination on the seasonal incidence of rotavirus diarrhoea. (*a*) Relative size of peak seasonal incidence (as indicated by the colour bar) 5 years after vaccine introduction compared with peak incidence prior to vaccination for vaccine coverage levels of 50, 70 and 90 per cent of all infants at four months of age. (*b*,*c*) Weekly (blue lines) and average annual incidence (red lines) of severe rotavirus diarrhoea for a country with (*b*) CBR = 20 live births per 1000 and *R*_0_ = 35, and (*c*) CBR = 15 live births per 1000 and *R*_0_ = 25. Vaccination is introduced in year 3 at (i) 50%, (ii) 70% and (iii) 90% coverage.

Since vaccination roughly equates to a decline in the birth rate by reducing the recruitment of fully susceptible individuals and a reduction in the transmission rate by preventing the most infectious primary cases, we might predict from figure 2 that vaccination could increase the seasonality of epidemics under certain circumstances. Vaccination led to a decrease in the average annual incidence of severe diarrhoea predicted by the model that was comparable across all birth rates and transmission rates we explored. Under most circumstances, vaccination was also expected to decrease the peak incidence of severe diarrhoea in a typical post-vaccination year (figure 3). However, for certain combinations of birth rates and transmission rates, vaccination could indeed lead to an *increase* in the peak incidence of severe diarrhoea occurring at certain times of the year as the dynamics transitioned to a pattern of more seasonal epidemic disease or biennial epidemics. This is illustrated for the case of a country with an intermediate birth rate (CBR = 20 live births per 1000) and transmission rate (*R*_0_ = 35) with a vaccine coverage of 50 per cent (figure 3*b*). As vaccine coverage increased, the relative peak incidence tended to decrease across all combinations of birth rates and transmission rates, but countries with higher birth rates and/or transmission rates became more at risk for increased peak seasonal incidence (figure 3*a*). Furthermore, there may be a transition period soon after vaccine introduction in which seasonal incidence is more pronounced.

Those countries most at risk for increased peak incidence following the introduction of rotavirus vaccination are ones with intermediate birth rates and transmission rates. Given our estimates of *R*_0_ for the representative countries with published age distributions, this may include countries such as Uzbekistan. Countries such as Cambodia and Nepal with slightly higher birth rates and transmission rates could experience similar phenomena as vaccine coverage levels reach 90 per cent or more, with a vaccine efficacy of 80 per cent. However, it may be difficult to achieve this level of effective coverage if the vaccine is less efficacious in such settings.

**Figure 4.**
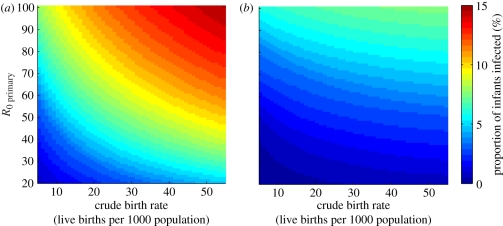
Predicted age of first infection with rotavirus. Proportion of infants infected with rotavirus prior to 4 months of age (indicated by the colour bar) across a range of values for the CBR and transmission rate (*R*_0_) for the periods (*a*) prior to vaccine introduction, and (*b*) 10 years after vaccine introduction with 90% coverage.

The proportion of infants infected prior to four months of age also showed a strong dependence on both the birth rate and the transmission rate (figure 4). At high birth rates and transmission rates, up to 12 per cent of infants may be infected with rotavirus prior to four months of age, which is the recommended age at which the second dose of rotavirus vaccine is administered in most developed countries (figure 4*a*). Thus, vaccination may not have as great an impact initially in such settings if vaccination is not as effective in previously infected infants. However, vaccination is expected to lead to a decrease in the force of infection (i.e. incidence rate per susceptible individual) and thus a delay in the time to infection, such that the proportion of infants infected prior to 4 months of age decreased substantially at high levels of vaccine coverage (figure 4*b*). This could lead to an increase in vaccine impact over time under these assumptions.

## 4. Discussion

Much of the promise of rotavirus vaccines lies in their ability to reduce the incidence of severe disease in developing countries, where diarrhoea is still a major cause of both morbidity and mortality [1,2]. Determining how the potential impact of vaccination may vary between developed versus developing countries requires an understanding of how the transmission dynamics of rotavirus differ in these settings. Birth rates have been shown to be an important determinant of the spatio-temporal pattern of rotavirus epidemics in the USA [13], and may also help explain why rotavirus tends to be less seasonal in developing countries, which often exhibit high birth rates and high transmission rates. Using a mathematical model fitted to epidemiological data from 15 countries, we show that geographical differences in birth and transmission rates can partially explain the observed variation in rotavirus seasonality. Our model also suggests that vaccination could have the unexpected consequence of increasing the seasonality of rotavirus disease in countries with intermediate-to-high birth rates and/or transmission rates while still reducing the mean annual incidence of disease.

While the combination of birth rates and transmission rates are likely important determinants of the magnitude of seasonal variation and can affect small differences in the timing of epidemics, these factors do not explain why rotavirus tends to occur more frequently during the cool, dry season in both temperate and tropical regions [20,22,23]. Environmental factors such as temperature and/or humidity probably play a role in organizing the peak of rotavirus transmission to times when conditions are most favourable to virus survivability and transmission [25–28]. However, even small seasonal differences in the transmission rate can resonate with the epidemic clockwork to produce large seasonal epidemics evident in some countries. It should be noted that these large seasonal epidemics are not confined to temperate regions of the world, as previously thought [20]. Pronounced seasonality has been observed in subtropical and tropical countries, such as Hong Kong and Taiwan (since 2005) [44,48], as well as regions of Venezuela and Brazil [52,53]. By contrast, temperate countries such as Uzbekistan exhibit only weak seasonal variation in rotavirus incidence [41]. The combination of lower birth rates and/or lower transmission rates may help in explaining why rotavirus tends to be moderately to strongly seasonal in countries such as Hong Kong and the Lao PDR, but is only weakly seasonal in neighbouring Cambodia (table 1 and electronic supplementary material, figure S1) [36,44,45]. Our results also indicate that infection tends to occur at an earlier age when birth rates and transmission rates are high. Thus, the observed association between year-round circulation of rotavirus and a lower average age of cases may be confounded by the common causes of high birth rates and/or transmission rates rather than being indicative of a causal relationship between seasonality and age of infection [53,54].

The effect of vaccination is dynamically similar to a reduction in the birth rate since the number of fully susceptible infants entering the population is decreased and a reduction in the transmission rate since it prevents the most infectious primary cases [13]. This helps explain why epidemics occurred later and with lower amplitude following the introduction of rotavirus vaccination in countries such as the USA and Mexico [18,19,55], since vaccination led to a delay in the build-up of a sufficient number of *susceptible* infants and highly infectious primary cases to set off an epidemic. Our model suggests vaccination may alter the dynamics of rotavirus in countries with intermediate birth rates and transmission rates, leading to a pattern of more seasonal epidemic disease. While the overall annual incidence of rotavirus diarrhoea is expected to decrease, peak seasonal incidence of rotavirus could actually increase in such settings. It is important to anticipate these changes in the dynamics, particularly if hospitals are not equipped to handle the increased number of diarrhoea admissions at the peak of the epidemic. This could also impact cost-effectiveness analyses, since the cost of hospitalization may increase, for example, if there is a shortage of beds. Finally, it will be important to evaluate the success of rotavirus vaccination in terms of the reduction in annual morbidity and mortality measures rather than comparing monthly indicators to their pre-vaccination levels. The occurrence of epidemics following the introduction of rotavirus vaccination to a country should not be considered indicative of vaccine failure. To avoid such effects, countries with intermediate birth rates and transmission rates most at risk for increased seasonal incidence may want to ensure that high levels of vaccine coverage are reached as quickly as possible after vaccine introduction.

In developing countries with high birth rates and transmission rates, achieving high coverage with a fully effective vaccine may not be as feasible. In recent clinical trials, the efficacy of rotavirus vaccination was lower in some developing country settings [4,6,10]. There are a number of possible explanations for this reduced efficacy related to host and environmental factors, including higher rates of breastfeeding and higher levels of transplacental maternal antibodies, co-administration of oral polio vaccine, interference by other diarrhoeal pathogens, micronutrient malnutrition and greater diversity of rotavirus strains in developing countries [56]. Furthermore, the high transmission rate and year-round circulation of rotavirus in such settings may lead to infection prior to enrolment in vaccine trials and/or the administration of all vaccine doses, as we show here. This could contribute to lower efficacy estimates if the effect of vaccination is reduced in previously infected individuals, although it is unlikely to explain all of the observed effect. Nevertheless, even moderately efficacious vaccines can prevent considerable numbers of hospitalizations and deaths due to rotavirus in these high burden settings [4,6,10]. If vaccination is introduced in such countries at appreciable coverage levels, it is possible that the observed vaccine effectiveness may actually *increase* over time as the force of infection is reduced, thereby leading to a delay in the time to first infection; this is an important consequence of the ‘herd immunity’ generated by vaccination. Furthermore, if mothers in developing countries tend to have higher maternal antibody titres as a result of continual boosting by exposure to rotavirus, then this reduction in the force of infection may also decrease the neutralizing effect these maternal antibodies could be having on the vaccine when administered to infants.

The fit of our model to the age distribution of cases in different countries reveals an element of added complexity in the nature of maternal immunity and protection against rotavirus. The strength and duration of maternal immunity appear to be greater than previously assumed in models of rotavirus transmission [11,13,16]. This is particularly evident in the age distributions of cases from developing countries, where the greatest proportion of cases occurs in the six to 11 months age group, but the zero to five months age group is comparatively underrepresented. It is possible that maternal immunity tends to be long-lasting in developing countries compared with developed countries because of the combination of higher rates and longer duration of breastfeeding, and possible immune boosting in mothers resulting from more recent re-infection (and/or recent infection with a greater diversity of strains). Further exploration of these differences is warranted as it could help explain the reduced efficacy of vaccination in developing countries.

Surveillance studies conducted in anticipation of rotavirus vaccination have yielded descriptions of the age distribution and seasonal pattern of rotavirus-associated diarrhoea in children less than 5 years of age from a variety of different countries. We used these age distributions to estimate the transmission rate of rotavirus in these settings given the birth rate is known; our estimates range between *R*_0_ = 28 and *R*_0_ = 191. These estimates should not be over-interpreted and will probably be biased in some instances. Other factors are likely to affect the reported age distribution of rotavirus cases from which these estimates were derived, such as differences in treatment-seeking behaviour, the prevalence of co-infections which may affect rotavirus testing rates, mortality patterns and other population demographics, infant care practices, population mixing, etc. Furthermore, surveillance methods differed from study to study. While some studies only tested patients admitted to the hospital or clinic, others tested both inpatients and outpatients; there may be differences in the age distribution and reporting rate of severe rotavirus cases compared with those with more moderate diarrhoea. Greater representation of moderate diarrhoea cases in older age individuals could lead to underestimation of *R*_0_ in studies that included outpatients. The duration of surveillance also differed among studies, and the age distribution and seasonality of rotavirus diarrhoea in a given year may not be indicative of long-term patterns.

Our modelling assumptions will also affect our estimates of the transmission rate. For example, we assumed mixing is homogeneous, but other population mixing assumptions may lead to lower estimates of *R*_0_ (see the electronic supplementary material). Also, we assumed that birth rates and transmission rates were constant over time when in fact they are likely to have changed. Our model is in part based on estimates of natural immunity from infection derived from a cohort study conducted in Mexico [57]. While other studies have demonstrated similar findings [58,59], the strength of natural immunity from previous rotavirus infections may not be the same in all settings, and in particular may be weaker in some developing countries. Furthermore, we assumed that disease severity is related to the number of previous infections rather than the age of infected individuals; it is very difficult to disentangle these two highly correlated effects. If rotavirus infections tend to be less severe in older children *independent* of the number of previous infections, then we may be overestimating the transmission rate in some settings, as well as underestimating the impact of vaccination [13].

Nevertheless, the estimates of the transmission rate which we derive are useful in that they allow us to compare patterns of rotavirus seasonality predicted by the model for different settings using very limited data. More reliable estimates of the transmission rate, as well as estimates of seasonal variation in the transmission rate and underreporting factors, can and should be obtained by fitting models to age-stratified time series of rotavirus cases from specific settings. Anticipating the country-specific impact of vaccination requires an understanding of how vaccine effectiveness and the transmission dynamics of rotavirus may vary among settings and over time.

Determining how and why seasonal patterns of rotavirus-associated diarrhoea differ between developed and developing countries is an important element in evaluating how the impact of vaccination observed presently in the Americas, Australia and Europe may translate to the context of developing countries [3]. The relationship we describe between birth rates, transmission rates and the degree of seasonal fluctuation in rotavirus incidence predicted by our model helps to broaden this understanding. However, other factors remain unexplained, such as what are the environmental and other sources of seasonal forcing that lead to rotavirus being more common in the cool, dry season in most parts of the world? Additionally, why is a greater diversity of rotavirus strains observed in developing countries compared with developed countries, and how will this impact vaccine effectiveness? Mathematical modelling of the transmission dynamics of rotavirus may help to address some of these questions, but further epidemiological and experimental studies are essential in improving our understanding of these issues and will help to inform future models for rotavirus dynamics.
